# Incremental Pulse-Width Erase (IPWE) Scheme for Fast and Variation-Tolerant GIDL Erase of 3D NAND Flash

**DOI:** 10.3390/mi17040399

**Published:** 2026-03-25

**Authors:** Youngjun Park, Wonbo Shim

**Affiliations:** Department of Electrical and Information Engineering, Seoul National University of Science and Technology, Seoul 01811, Republic of Korea; youngjunpark@seoultech.ac.kr

**Keywords:** 3D NAND flash memory, CD variation, cell on peripheral, GIDL erase, temperature variation

## Abstract

In this work, we propose an incremental pulse-width erase (IPWE) scheme for fast and variation-tolerant gate-induced drain leakage (GIDL) erase of 3D NAND flash. For the GIDL erase operation, GIDL-generated hole accumulation is required to raise the channel potential. This requirement leads to a transient state that degrades erase speed and broadens distribution of the erased Vth. In addition, the degradation becomes more pronounced with critical-dimension (CD) variation and temperature variation. The proposed IPWE scheme increases erase pulse width progressively, rather than increasing erase voltage as in the conventional incremental step pulse erase (ISPE) scheme. Sentaurus TCAD simulations of a 3D NAND flash with a surrounded BL PAD structure demonstrate that the IPWE scheme achieves a 1.18 V larger Vth shift compared to the ISPE scheme for the same total erase time of 6.6 ms. The IPWE scheme also effectively narrows the erase Vth shift distribution, reducing it by 40 mV under a 55 nm CD variation, 0.26 V for a 10 nm CD variation between channel strings, and 2 V across a 50 K temperature variation, all within a total erase time of 6.6 ms.

## 1. Introduction

The rapid expansion of artificial intelligence and cloud computing has accelerated the demand for high-density nonvolatile memory (NVM), driving the development of various NVM technologies [1,2,3,4,5]. In particular, 3D NAND flash memory requires further scaling. To improve the bit density of 3D NAND flash memory, the cell over peripheral (COP) architecture has been introduced, where peripheral circuits and page buffers are placed underneath the cell array [6,7,8,9]. While the COP architecture effectively increases array density, it requires peripheral circuits on the silicon substrate. Accordingly, structures such as the channel hole sidewall ONO butting (CSOB) structure are required, where the cell array is formed on an N^+^ poly-Si layer electrically isolated from the Si substrate [10]. Unlike the conventional 3D NAND architecture based on the body contact spacer (BCS) structure, CSOB structure cannot employ substrate-driven bulk erase. This is because the channel is electrically isolated from the substrate, whereas bulk erase in the BCS structure can be realized by directly applying the erase bias to the substrate. Therefore, the CSOB structure employs the GIDL current to enable the erase operation.

For the GIDL erase [11] operations, holes generated by GIDL must be accumulated in the channel until the channel potential rises sufficiently for the erase operation, which could cause a delay before effective erase. As the number of word-line (WL) layers increases, the volume and capacitance of the channel become larger, requiring more accumulated holes to elevate the channel potential. Consequently, the channel potential rise rate decreases and the erase speed may be degraded. To address this limitation, a surrounded BL PAD structure was proposed to enlarge the GIDL generation region and substantially increase hole generation, thereby accelerating channel potential rise [12,13]. In contrast to such structural innovations, the erase bias scheme still employs an incremental step pulse erase (ISPE) scheme, in which the erase pulse amplitude is increased while the pulse width is kept constant.

**Figure 1:**
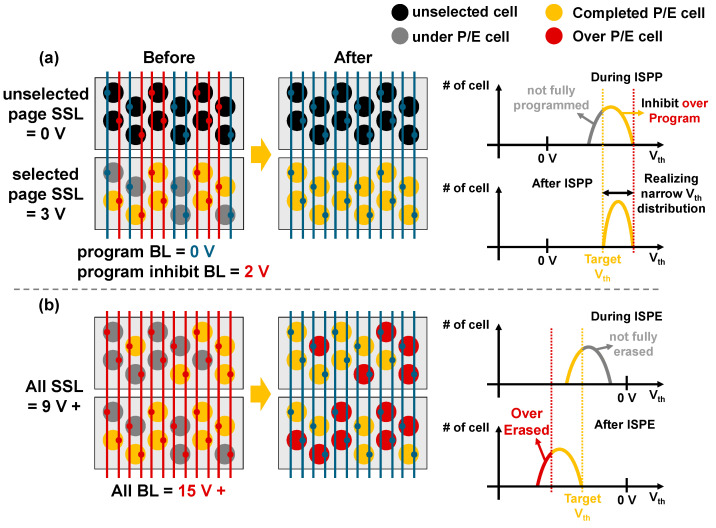
Top view of a 3D NAND flash memory array and cell distribution plots (**a**) during the program operation and (**b**) during the erase operation.

To ensure device retention and endurance, reducing the threshold voltage (Vth) distribution after program and erase operations is essential. However, process variations in 3D NAND fabrication cause the Vth distribution to widen. Because the program operation is performed at the page level, each BL can effectively control a single selected channel string, enabling program inhibit schemes during incremental step pulse programming (ISPP) to prevent over programming of cells that have already reached the target threshold voltage (Vth) as illustrated in Figure 1. In contrast, erase operation is executed at the block level. Therefore, unlike the program operation, the erase operation cannot employ an inhibit scheme, leading to the formation of over-erased cells. Over erase degrades both endurance and retention, thereby requiring additional corrective techniques, such as the deep erase compensation (DEC) scheme [14]. Therefore, it is essential to reduce Vth distribution and minimize the number of over-erased cells.

In this work, we propose the incremental pulse-width erase (IPWE) scheme for fast and variation-tolerant GIDL erase of 3D NAND flash with the surrounded BL PAD structure. Unlike the conventional ISPE scheme, the IPWE scheme improves erase speed and reduces Vth distribution by increasing the erase pulse width rather than the erase pulse voltage. The effectiveness of the proposed scheme was evaluated by Sentaurus TCAD simulation.

## 2. Device Structure and Simulation Methodology

**Figure 2:**
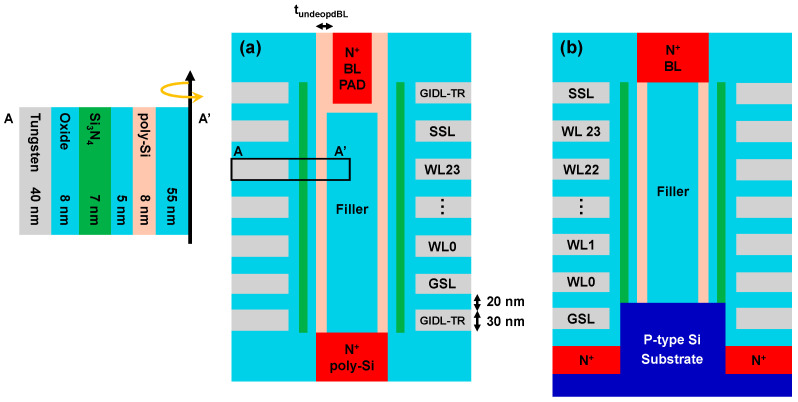
Architecture cross-sectional views of (**a**) 3D NAND string with surrounded BL PAD structure for GIDL erase and (**b**) 3D NAND string with SEG structure for bulk erase.

As shown in Figure 2, we designed a 3D NAND string with the surrounded BL PAD structure for GIDL erase to investigate the effectiveness of the IPWE scheme. The channel is connected to a N^+^ poly-Si plate doped at 1 × 10^20^ cm^−3^. To increase the hole generation rate, GIDL transistors (GIDL-TR) were incorporated above the SSL and below the GSL. In addition, we designed a 3D NAND string with selective epitaxial growth (SEG) structure for bulk erase case to compare the channel potential between GIDL erase and bulk erase.

The gate stack comprised an 8 nm blocking oxide, a 7 nm silicon nitride charge trap layer, and a 5 nm tunneling oxide. The polysilicon channel thickness and filler diameter were set to 8 nm and 110 nm, respectively. The vertical dimensions were set with a WL length of 30 nm and a spacer length of 20 nm. Device simulations employed the Philips unified mobility (PhuMob) model and a high-field saturation mobility model. Doping-dependent SRH recombination, Auger recombination, and band to band tunneling (BTBT) modeled using the Hurkx approach were also included.

Table 1 and Table 2 summarize the bias conditions for the program, erase, and read operations. For the program operation, the operation timing consists of a 30 µs execution phase, with ramp-up and ramp-down times of 1 µs each. For the erase operation, each pulse included a 100 μs ramp-up and a 1 μs ramp-down, and the initial erase pulse width was set to 1 ms.

The surrounded BL PAD structure is implemented to accelerate the channel potential rise by enlarging the GIDL generation region [12]. The structure consists of an undoped BL region surrounding a doped N^+^ poly-Si BL, and the BL PAD thickness determines the GIDL erase speed. We designed the structure with a 45 nm undoped BL PAD thickness (t_undopedBL_), which yields the fastest channel potential rise.

## 3. Incremental Pulse-Width Erase (IPWE)

**Figure 3:**
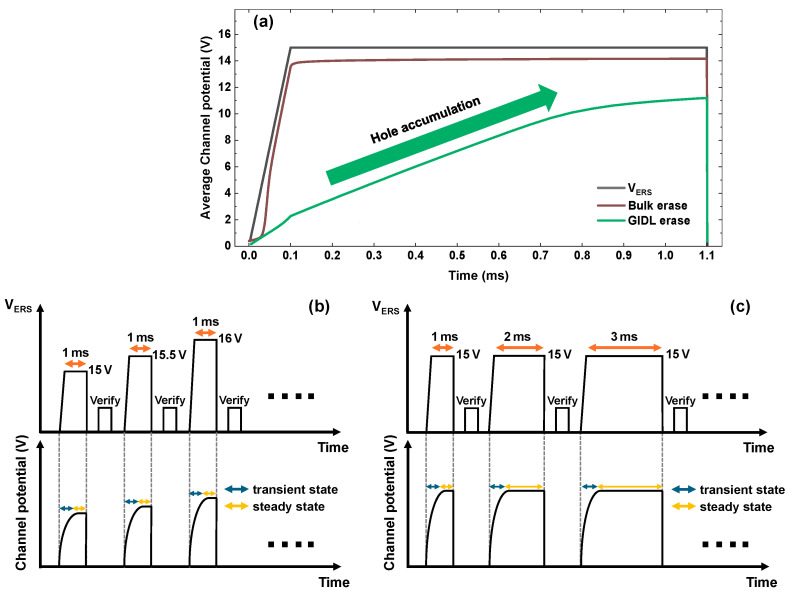
V_ERS_ pulse and average channel potential for (**a**) a comparison between bulk erase and GIDL erase, (**b**) the conventional ISPE scheme, and (**c**) the proposed IPWE scheme.

Bulk erase directly injects holes from the p-type substrate into the channel. Therefore, the channel potential rises and completes its increase simultaneously with the ramp-up of $V_{ERS}$. However, in GIDL erase, a transient state is required for GIDL-generated holes to accumulate in the channel until the Fowler–Nordheim (FN) tunneling current becomes sufficient to erase the cells, as shown in Figure 3a. As illustrated in Figure 3b,c, the ISPE scheme maintains a constant erase pulse width. In contrast, the IPWE scheme progressively increases the erase pulse width as the erase operation proceeds. Consequently, in later pulses, the IPWE scheme reduces the fraction of the transient state in the total erase time compared with the ISPE scheme. Thus, the fraction of the steady state in the total erase time increases. Therefore, the time in a high-voltage state (t_high_) sufficient to induce Fowler–Nordheim (FN) tunneling also increases.

### 3.1. Erase Speed Enhancement

**Figure 4:**
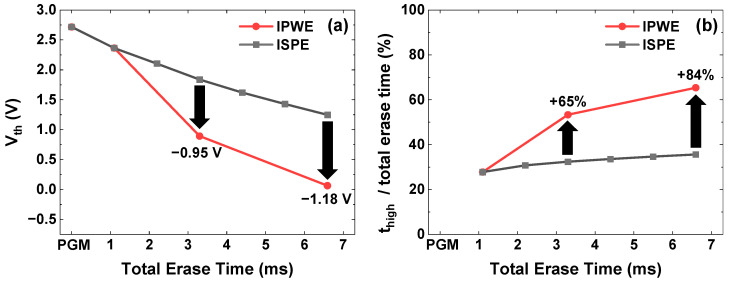
(**a**) Comparison of the Vth shift with the conventional ISPE scheme and the proposed IPWE scheme. (**b**) Percentage of t_high_ (duration with channel potential > 7.5 V) within the total erase time.

At the first erase pulse, Figure 4a shows that both schemes exhibit the same Vth shift because the same erase pulse is applied. However, starting from the second erase pulse, the IPWE scheme exhibits a 0.95 V larger Vth shift than the ISPE scheme because t_high_ increases within the same total erase time, as shown in Figure 4b. t_high_ is defined as the duration for which the channel potential is above 7.5 V, which is half of the initial V_ERS_. Due to the continued increase in t_high_ in subsequent erase pulses, the IPWE scheme achieves a 1.18 V larger Vth shift than the ISPE scheme after a total erase time of 6.6 ms.

### 3.2. Reduction in Vth Distribution Under CD Variation

**Figure 5:**
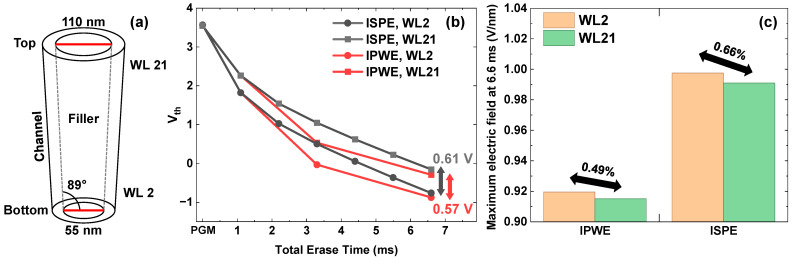
(**a**) Taper angle and top and bottom diameters of the filler. (**b**) Comparison of Vth between the upper WL (WL21) and lower WL (WL2) using the ISPE scheme and IPWE schemes. (**c**) Difference in the maximum electric field across the tunneling oxide between WL2 and WL21 at a total erase time of 6.6 ms.

In 3D NAND fabrication, as illustrated in Figure 5a, channel holes exhibit a taper angle due to fabrication difficulties during high aspect ratio etching processes [15], resulting in different channel hole diameters at upper and lower WL positions. This CD variation leads to different electric fields according to the location of WLs. Consequently, even within the same channel string, the erase Vth shift varies between upper and lower WLs. Figure 5b shows that the erase Vth shift difference between the upper and lower WLs is smaller with the proposed IPWE scheme than with the conventional ISPE scheme. The IPWE scheme exhibited a 40 mV smaller Vth shift difference at a total erase time of 6.6 ms. As shown in Figure 5c, this result arises because the IPWE scheme applies a lower erase voltage than the ISPE scheme, thereby reducing the difference in the electric field across the tunneling oxide between WL21, which is located in the upper region of the channel hole, and WL2, which is located in the lower region of the channel hole.

**Figure 6:**
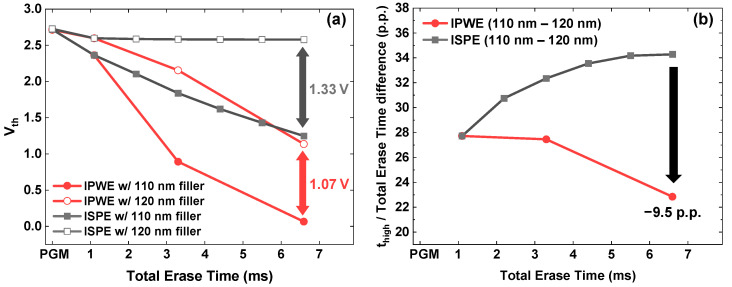
Comparison of the IPWE and ISPE schemes. (**a**) Vth shift at filler diameter of 110 nm and 120 nm. (**b**) Difference in the percentage of t_high_ within the total erase time induced by CD variation (percentage points, p.p.).

Additionally, CD variation among channel strings leads to differences in channel volume between channel strings. Therefore, even at the same WL position, different channel strings can exhibit different erase Vth shifts. As shown in Figure 6a, the IPWE scheme reduces the erase Vth shift distribution caused by CD variation across the 110 nm and 120 nm filler diameter from 1.33 V to 1.07 V compared with the ISPE scheme while achieving a larger erase Vth shift.

This difference originates from the change in t_high_ caused by CD variation. Due to volume differences among channel strings, channel potential rising speed varies from one channel string to another, which leads to Vth distribution. As described above, the IPWE scheme reduces transient state relative to the total erase time. Therefore, as shown in Figure 6b, the IPWE scheme exhibits a smaller change in t_high_ induced by CD variation than the ISPE scheme. This leads to a reduced Vth shift distribution.

### 3.3. Reduction in Vth Distribution Under Temperature Variation

**Figure 7:**
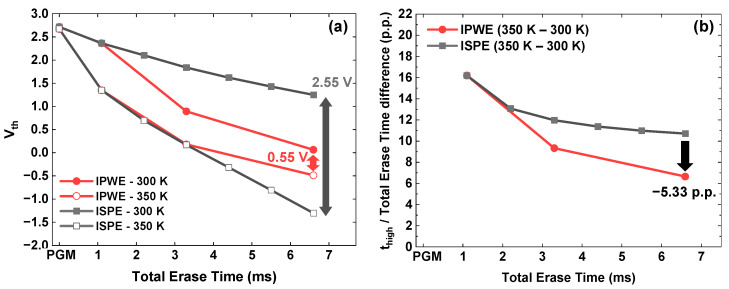
Comparison of the IPWE and ISPE schemes under temperature variation. (**a**) Vth shift at 300 K and 350 K. (**b**) Difference in the percentage of t_high_ within the total erase time induced by temperature variation (percentage points, p.p.).

As the operating temperature increases, bandgap narrowing in the poly-Si BL and channel enhances GIDL generation, resulting in a larger Vth shift in erase operation. This increased GIDL generation can broaden the Vth distribution. As shown in Figure 7a, increasing the temperature from 300 K to 350 K during the erase operation produces a Vth shift difference. The ISPE scheme exhibits a 2.55 V difference, whereas the IPWE scheme shows a much smaller difference of 0.55 V. This is because the IPWE scheme reduces the transient state relative to the total erase time. Therefore, the IPWE scheme exhibits a smaller change in t_high_ induced by temperature variation than the ISPE scheme, as shown in Figure 7b. This leads to a reduced Vth shift distribution.

## 4. Conclusions

In this study, we proposed an incremental pulse-width erase (IPWE) scheme for fast and variation-tolerant GIDL erase of 3D NAND flash. Increasing the erase pulse width reduces the fraction of the transient state in the total erase time compared with the conventional ISPE scheme. This reduction enhances erase speed and achieves a narrower erase Vth distribution under channel hole diameter variations and temperature variations. In addition, the reduced electric field enabled by a lower erase voltage narrows the Vth distribution under taper angle variation in a single channel string. These characteristics of the IPWE scheme demonstrate its potential as an effective erase operation strategy for high-stack COP architecture 3D NAND flash memory, where the increased channel volume leads to a slower rise in channel potential during the erase operation, while CD variation becomes more pronounced. In addition, the proposed IPWE scheme offers a practical bias-engineering approach that improves erase performance without requiring structural modification of the device, thereby minimizing additional process complexity and fabrication cost. Future work could investigate the IPWE scheme with additional non-ideal channel structures, such as bowing and non-circular channel string profiles, as well as its retention and endurance characteristics.

## Figures and Tables

**Table 1:** Bias conditions at program, erase, and read operations of surrounded BL PAD structure.

Contact	Program	Erase (GIDL)	Read
BL	0 V	V_ERS_	0.2 V
CSL	0 V	V_ERS_	0 V
SSL	2 V	V_ERS_ − 6 V	7 V
GSL	0 V	V_ERS_ − 6 V	7 V
GIDL-TR	2 V	V_ERS_ − 6 V	7 V
Selected WL	V_PGM_	0 V	V_Read_
Unselected WLs	7 V	0 V	7 V

**Table 2:** Bias conditions at program, erase, and read operations of SEG structure.

Contact	Program	Erase (Bulk)	Read
BL	0 V	V_ERS_	0.2 V
CSL	0 V	floating	0 V
SSL	2 V	floating	7 V
GSL	0 V	V_ERS_ − 6 V	7 V
Substrate	0 V	V_ERS_	0 V
Selected WL	V_PGM_	0 V	V_Read_
Unselected WLs	7 V	0 V	7 V

## Data Availability

The data presented in this study are available on request from the corresponding author. The data are not publicly available due to research security.
